# The relationship between non-high-density lipoprotein cholesterol to high-density lipoprotein cholesterol ratio and abdominal aortic calcification in adults: a cross-sectional study

**DOI:** 10.3389/fcvm.2025.1578407

**Published:** 2025-11-17

**Authors:** Jiawei Zhang, Yijie Ning, Rui Bai, Liying Song, Hongqin Wang

**Affiliations:** 1 Department of Neurosurgery, The First Hospital of Shanxi Medical University, Taiyuan, China; 2 Department of Vascular Surgery, The Second Hospital of Shanxi Medical University, Taiyuan, China; 3 Vascular Institute of Shanxi Medical University, Taiyuan, China; 4 Department of Thyroid Surgery, First Hospital of Shanxi Medical University, Taiyuan, China

**Keywords:** NHHR, abdominal aortic calcification, cross-sectional study, NHANES, RCS

## Abstract

**Background:**

The non-high-density lipoprotein cholesterol to high-density lipoprotein cholesterol ratio (NHHR) is a novel lipid index for assessing atherosclerosis. Although NHHR has been recognized as a biomarker for multiple diseases, its association with abdominal aortic calcification (AAC) remains unexplored.

**Methods:**

This study analyzed data from 2,517 participants in the 2013 to 2014 National Health and Nutrition Examination Survey (NHANES). AAC was assessed using dual-energy x-ray absorptiometry and quantified with the Kauppila score (AAC−24). The relationship between NHHR and AAC was evaluated using multivariate linear and logistic regression models, with nonlinear associations visualized via restricted cubic splines. Subgroup and interaction analyses were conducted to assess the robustness of the findings across different populations.

**Results:**

In fully adjusted models, AAC scores and severe AAC (sAAC) prevalence increased with each quartile increment of NHHR (*p* < 0.05). A one-unit increase in NHHR was associated with a 0.13-unit rise in AAC score (*β* = 0.13, 95% CI: 0.02–0.24) and a 19% increase in sAAC risk (OR = 1.19, 95% CI: 1.02–1.40). Subgroup analysis identified a significant interaction between NHHR and gender in relation to AAC. The OR (95% CI) was 0.97 (0.77–1.23) in males and 1.46 (1.18–1.81) in females (*p* for interaction = 0.008).

**Conclusion:**

In adults aged 40 years and older, higher NHHR levels were associated with increased AAC scores and a greater risk of sAAC, particularly among women. Furthermore, this study highlights the potential clinical value of NHHR in the prevention of AAC and its related complications.

## 1 Introduction

Abdominal aortic calcification (AAC) is a pathological progression actively regulated in response to local or systemic environmental disturbances (1). It is characterized by abnormal deposition of calcium phosphate crystals on the inner surface of blood vessels and the intimal layer (2). AAC is strongly associated with aging, smoking, metabolic disorders, and kidney disease (1). Both coronary artery calcification and AAC are manifestations of vascular calcification, with AAC being closely linked to cardiovascular disease mortality and all-cause mortality (3). Furthermore, AAC serves as a significant predictor of myocardial infarction (3), stroke (4), and congestive heart failure (5). The incidence of these conditions increases significantly with the severity of AAC.

Blood lipids play a crucial role in atherosclerosis formation, making their involvement in arterial calcification significant. In recent times, the non-high-density lipoprotein cholesterol to high-density lipoprotein cholesterol ratio (NHHR) has gained attention as a novel index for predicting the risk of cardiovascular events (6). Studies have reported an association between NHHR and various long-term illnesses, including diabetes (7), fatty liver illnesses (8), metabolic syndrome (9), and kidney stones (10). Additionally, research indicates that AAC is associated with cholesterol (*p* < 0.05), high-density lipoprotein (HDL-C) (*p* < 0.01), and low-density lipoprotein cholesterol (LDL-C) (*p* < 0.05) (11). Furthermore, a previous study (12) confirmed that HDL-C is negatively correlated with aortic calcification. Integrating NHHR with other atherogenic and anti-atherosclerotic indicators holds promise for predicting the severity of AAC and preventing cardiovascular events. Accordingly, this study conducted a cross-sectional analysis to investigate the association between NHHR and AAC, assessing whether higher NHHR levels are associated with increased AAC scores and a greater risk of severe AAC in adults aged 40 years and older.

## 2 Methods

### 2.1 Data sources

The data were obtained from the National Health and Nutrition Examination Survey (NHANES), a cross-sectional survey designed to assess the health status of the U.S. population. NHANES employs a probability-based multistage sampling design to ensure a nationally representative sample of the non-hospitalized civilian population in the United States. Conducted every two years, the survey collects health information through standardized protocols. The NHANES survey protocol was approved by the Research Ethics Review Board of the National Center for Health Statistics, and all participants provided written informed consent.

### 2.2 Assessment of NHHR and AAC

This study analyzed the NHHR in plasma as the primary exposure variable. NHHR is the ratio of total cholesterol minus high-density cholesterol (non-HDL-C) to high-density cholesterol (HDL-C). NHHR was analyzed primarily as a continuous variable and secondarily as a categorical variable divided into quartiles.

AAC served as the dependent variable. NHANES provides AAC data obtained through dual-energy x-ray absorptiometry (DXA), with strict quality control measures applied to data collection and scan analysis. The University of California, San Francisco, evaluates participant scans using standard radiology protocols tailored for NHANES. The Kauppila method (AAC-24 score) was used to assess AAC severity. The grading criteria for aortic calcification in each segment are detailed in Supplementary Table 1. Based on existing research, a Kauppila score greater than 6 was classified as severe AAC (sAAC). Participants included in the final analysis were categorized into two groups: “no severe AAC (AAC score ≤ 6)” and “sAAC (AAC score >6).”

### 2.3 Covariates

Covariates included demographic characteristics [sex, age, marital status, race, educational level, and the poverty income ratio (PIR)]; lifestyle risk factors [smoking and drinking status]; physical examination data [body mass index (BMI), systolic blood pressure (SBP), and diastolic blood pressure (DBP)]; comorbidities [hypertension, diabetes, heart failure, coronary heart disease (CHD), angina, heart attack, and stroke history]; biochemical indicators [glycohemoglobin, total cholesterol (TC), and high-density lipoprotein cholesterol (HDL)]; renal function biomarkers [uric acid and creatinine levels]; and bone mineral metabolism markers [25-hydroxyvitamin D, serum calcium, and phosphorus levels]. All variables were obtained through household interviews and examinations at the Mobile Examination Center (MEC). Detailed measurement procedures are available at: https://www.cdc.gov/nchs/nhanes/.

The race variable included Mexican American, other Hispanic, non-Hispanic White, non-Hispanic Black, and other races. Smokers were defined as individuals who had smoked at least 100 cigarettes in their lifetime. Drinkers were defined as those who consumed alcoholic beverages more than 12 times per year. BMI was calculated as weight (kg) divided by height squared (m^2^). Hypertension was defined as a self-reported physician diagnosis, current use of antihypertensive medication, or measured blood pressure ≥140/90 mmHg. Diabetes was defined as a self-reported physician diagnosis, use of oral hypoglycemic agents or insulin, fasting plasma glucose ≥126 mg/dl, glycohemoglobin (HbA1c) ≥6.5%, or 2-h plasma glucose ≥200 mg/dl after an oral glucose tolerance test.

### 2.4 Statistical analysis

In this study, continuous variables were expressed as means and standard deviations, while categorical variables were presented as counts (*n*) and percentages (%). Comparisons of continuous variables were performed using the Kruskal–Wallis test, while categorical variables were analyzed using chi-square tests. The association between NHHR and AAC was evaluated using multivariate linear and logistic regression models, with the relationship expressed through β values, odds ratios (ORs), and corresponding 95% confidence intervals (95% CI). Three models were established: Model 1 was conducted without covariate adjustments, Model 2 adjusted for race, gender, BMI, and age, and Model 3 further adjusted for marital status, education, PIR, smoking history, drinking history, hypertension, diabetes, heart failure, CHD, angina, stroke, TC, creatinine, serum calcium, phosphorus, uric acid, total 25-hydroxyvitamin D, and glycated hemoglobin.

Drawing upon these analyses, differences in AAC scores and sAAC risk among NHHR quartile groups were further evaluated. Additionally, independent ordinal variables were used to assess linear trends in NHHR. Restricted cubic spline (RCS) models derived from Model 3 were applied to investigate potential nonlinear dependencies linking NHHR and both AAC scores and sAAC. Finally, to determine whether the relationship between NHHR and AAC scores or sAAC was influenced by age, gender, lifestyle factors, underlying diseases, or cardiovascular events, subgroup analyses were performed. Stratifications included age (<55 years vs. ≥55 years), gender (male vs. female), alcohol consumption (yes vs. no), diabetes (yes vs. no), coronary heart disease (yes vs. no), stroke (yes vs. no), and hypertension (yes vs. no). These analyses aimed to evaluate the stability of the NHHR-AAC relationship across subgroups and to detect potential interactions. All statistical analyses were conducted using R version 4.4.1 (R Foundation for Statistical Computing, Vienna, Austria). A *p*-value of <0.05 was considered statistically significant.

## 3 Result

**Figure 1:**
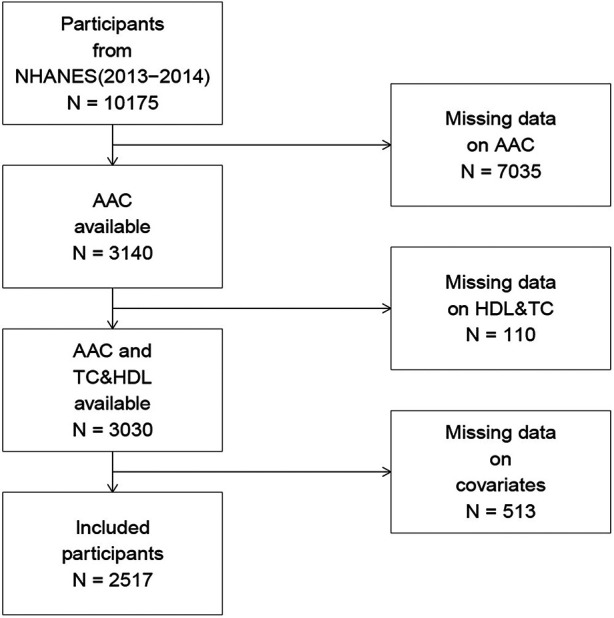
Flow chart of participant recruitment.

In the NHANES (2013–2014) cohort, 10,175 participants completed the interview, of whom 3,140 had valid AAC score data. Participants with missing AAC scores, TC, or HDL-C data (*n* = 110), along with those with missing covariate data (*n* = 513), were excluded. Ultimately, 2,517 participants were included in the final analysis. A comprehensive flowchart illustrating participant selection is presented in Figure 1.

### 3.1 Baseline

Table 1 summarizes the baseline characteristics of U.S. adults aged 40 years and older. Relative to participants without sAAC, individuals with sAAC exhibited a higher likelihood of being older and having a history of stroke, diabetes, hypertension, heart failure, and smoking. Additionally, these individuals had lower BMI, TC, and DBP but exhibited higher SBP glycated hemoglobin, creatinine, and 25-hydroxyvitamin D levels.

**Table 1:** Characteristics of study participants.

Characteristics	Level	Overall (*n* = 2,517)	No sAAC (*n* = 2,302)	sAAC (*n* = 215)	*p*
Gender	Male	1,226 (48.7%)	1,126 (49.3%)	100 (40.4%)	0.547^a^
Age (years)		58.94 ± 11.94	57.74 ± 11.48	71.80 ± 8.83	<0.001^b^
Marital status	Married/Living with partner	1,592 (68.4%)	1,485 (69.9%)	107 (48.9%)	<0.001^a^
	Widowed/Divorced/Separated	730 (24.3%)	630 (22.4%)	100 (49.1%)	
	Unmarried	195 (7.3%)	187 (7.7%)	8 (2.0%)	
Race	Mexican American	312 (6.6%)	295 (6.8%)	17 (4.2%)	<0.001^a^
	Other Hispanic	231 (4.3%)	222 (4.5%)	9 (1.7%)	
	Non-Hispanic White	1,172 (72.6%)	1,027 (72.0%)	145 (80.3%)	
	Non-Hispanic Black	483 (9.9%)	459 (10.1%)	24 (6.9%)	
	Other	319 (6.6%)	299 (6.6%)	20 (6.9%)	
Education level	Under high school	523 (14.6%)	470 (13.9%)	53 (22.8%)	0.227^a^
	High school or equivalent	565 (20.8%)	514 (20.8%)	51 (20.5%)	
	Above high school	1,429 (64.7%)	1,318 (65.3%)	111 (56.7%)	
PIR		2.71 ± 1.65	2.72 ± 1.66	2.61 ± 1.56	0.340^b^
BMI (kg/m^2^)		28.56 ± 5.61	28.68 ± 5.70	27.24 ± 4.31	<0.001^b^
Smoke	Yes	1,194 (46.5%)	1,060 (45.3%)	134 (62.2%)	<0.001^a^
Drink	Yes	1,829 (78.2%)	1,672 (78.5%)	157 (73.3%)	0.966^a^
SBP (mmHg)		127.35 ± 18.53	126.58 ± 18.19	135.54 ± 20.14	<0.001^b^
DBP (mmHg)		71.03 ± 11.55	71.69 ± 11.32	64.02 ± 11.63	<0.001^b^
Hypertension	Yes	1,375 (51.2%)	1,197 (48.9%)	178 (81.1%)	<0.001^a^
Diabetes	Yes	587 (19.6%)	505 (18.3%)	82 (35.8%)	<0.001^a^
Heart failure	Yes	85 (2.8%)	63 (2.4%)	22 (7.7%)	<0.001 ^a^
CHD	Yes	135 (4.7%)	90 (3.8%)	45 (17.2%)	<0.001^a^
Angina	Yes	81 (2.7%)	63 (2.4%)	18 (7.3%)	<0.001^a^
Heart attack	Yes	135 (4.8%)	103 (4.0%)	32 (15.6%)	<0.001^a^
Stroke	Yes	108 (3.7%)	84 (3.2%)	24 (10.3%)	<0.001^a^
Glycohemoglobin (%)		5.91 ± 1.15	5.89 ± 1.14	6.22 ± 1.27	<0.001
TC (mg/dl)		194.59 ± 42.80	195.70 ± 42.91	182.76 ± 39.87	<0.001^b^
HDL (mg/dl)		54.08 ± 16.88	54.17 ± 16.87	53.16 ± 16.96	0.404^b^
Uric acid (mg/dl)		5.45 ± 1.38	5.43 ± 1.38	5.62 ± 1.42	0.057^b^
Creatinine (mg/dl)		0.95 ± 0.54	0.94 ± 0.53	1.08 ± 0.63	<0.001^b^
Serum calcium (mg/dl)		9.46 ± 0.37	9.45 ± 0.37	9.49 ± 0.36	0.156^b^
Serum phosphorus (mg/dl)		3.79 ± 0.57	3.78 ± 0.57	3.91 ± 0.54	0.002^b^
Total 25-hydroxyvitamin D (nmol/L)		70.96 ± 29.52	70.00 ± 28.91	81.22 ± 33.77	<0.001^b^
NHHR		2.89 ± 1.40	2.91 ± 1.41	2.72 ± 1.27	0.055^b^
AAC-24 score		1.54 ± 3.32	0.67 ± 1.42	10.84 ± 3.55	<0.001^b^

PIR, household income-to-poverty ratio; BMI, body mass index; SBP, systolic blood pressure; DBP, diastolic blood pressure; CHD, coronary heart disease; TC, total cholesterol; HDL, high-density lipoprotein; AAC, abdominal aortic calcification.

a *P* value calculated using the Chi-square test.

b *P* value calculated using the Kruskal–Wallis test.

### 3.2 NHHR and AAC scores

Table 2 presents the relationship between NHHR and AAC scores, analyzed as both continuous and categorical variables. In Model 2, NHHR demonstrated a borderline significant positive correlation with AAC score (*β* = 0.09, 95% CI: −0.005 to 0.18, *p* = 0.063). In Model 3, NHHR was significantly associated with AAC score (*β* = 0.13, 95% CI: 0.02 to 0.24, *p* = 0.023). Each one-unit increase in NHHR was associated with a 0.13-unit higher AAC score. When NHHR was categorized into quartiles for analysis, the fully adjusted models showed that individuals within the top quartile exhibited a statistically significant elevation in AAC scores (*p* < 0.05) relative to the bottom quartile group. Specifically, the top quartile group had AAC scores 0.44 units higher than the bottom quartile group (*β* = 0.44, 95% CI: 0.03–0.85, *p* = 0.036), with a statistically significant trend (*p* for trend <0.05). (Results for additional variables are provided in Supplementary Tables 2–5.)

**Table 2:** Multivariate-adjusted β-values and 95% confidence intervals for the association between NHHR and AAC score.

NHHR level	β (95%CI)		
	Model 1	Model 2	Model 3
Continuous	−0.09 (−0.18 to –0.006) 0.067	0.09 (−0.005 to –0.18) 0.063	0.13 (0.02–0.24) 0.023
Q1	Reference	Reference	Reference
Q2	−0.11 (−0.47 to –0.26) 0.569	0.04 (−0.31 to –0.37) 0.828	0.10 (−0.24 to –0.43) 0.557
Q3	−0.24 (−0.61 to –0.12) 0.193	0.12 (−0.22 to –0.47) 0.490	0.21 (−0.14 to –0.57) 0.238
Q4	−0.24 (−0.61 to –0.12) 0.195	0.34 (−0.03 to –0.68) 0.064	0.44 (0.03–0.85) 0.036
*P*-trend	0.144	0.058	0.035

Model 1: no covariate adjustments.

Model 2: adjusted for age, sex, race, and BMI.

Model 3: further adjusted for marital status, education, PIR, smoking history, drinking history, hypertension, diabetes, heart failure, coronary heart disease, angina pectoris, stroke, total cholesterol, creatinine, serum calcium, phosphorus, uric acid, total 25-hydroxyvitamin D, and glycated hemoglobin, based on Model 2.

### 3.3 NHHR and sAAC

The multiple logistic regression analysis demonstrated a positive association between NHHR levels and sAAC risk (Table 3). When NHHR was analyzed as a continuous variable, it remained significantly associated with a higher risk of sAAC in both the partially adjusted Model 2 and the fully adjusted Model 3. Specifically, each one-unit increase in NHHR was associated with a 19% increase in sAAC risk. (OR = 1.19, 95% CI: 1.02–1.40, *p* = 0.023). This positive correlation persisted when NHHR was categorized into quartiles. Notably, in Model 3, participants in the top NHHR quartile had a 96% higher risk of sAAC compared to those in the bottom quartile (OR = 1.96, 95% CI: 1.10–3.48, *p* = 0.022; *p* for trend = 0.018). (Results for additional variables are presented in Supplementary Tables 7–9.)

**Table 3:** Multivariate-adjusted ORs and 95% confidence intervals for the association between NHHR and sAAC.

NHHR level	OR (95%CI)		
	Model 1	Model 2	Model 3
Continuous	0.90 (0.81–1.00) 0.055	1.10 (0.97–1.23) 0.124	1.19 (1.02–1.40) 0.023
Q1	Reference	Reference	Reference
Q2	0.91 (0.63–1.33) 0.630	1.14 (0.76–1.71) 0.512	1.34 (0.85–2.12) 0.204
Q3	0.76 (0.50–1.12) 0.164	1.16 (0.75–1.77) 0.506	1.61 (0.99–2.66) 0.061
Q4	0.75 (0.51–1.12)0.161	1.47 (0.95–2.29) 0.083	1.96 (1.10–3.48) 0.022
*P*-trend	0.103	0.105	0.018

Model 1: no covariate adjustments.

Model 2: adjusted for age, sex, race, and BMI.

Model 3: further adjusted for marital status, education, PIR, smoking history, drinking history, hypertension, diabetes, heart failure, coronary heart disease, angina pectoris, stroke, total cholesterol, creatinine, serum calcium, phosphorus, uric acid, total 25-hydroxyvitamin D, and glycated hemoglobin, based on Model 2.

### 3.4 Association between NHHR and AAC

**Figure 2:**
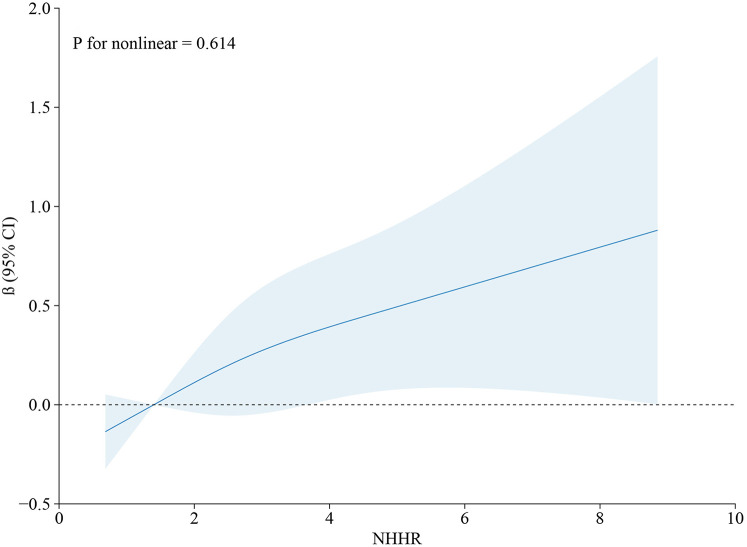
Restricted cubic spline model depicting the relationship between NHHR and AAC score. The restricted cubic spline model was adjusted for age, gender, race, BMI, marital status, education, PIR, smoking history, drinking history, hypertension, diabetes, heart failure, coronary heart disease, angina pectoris, stroke, total cholesterol, creatinine, serum calcium, phosphorus, uric acid, total 25-hydroxyvitamin D, and glycated hemoglobin.

**Figure 3:**
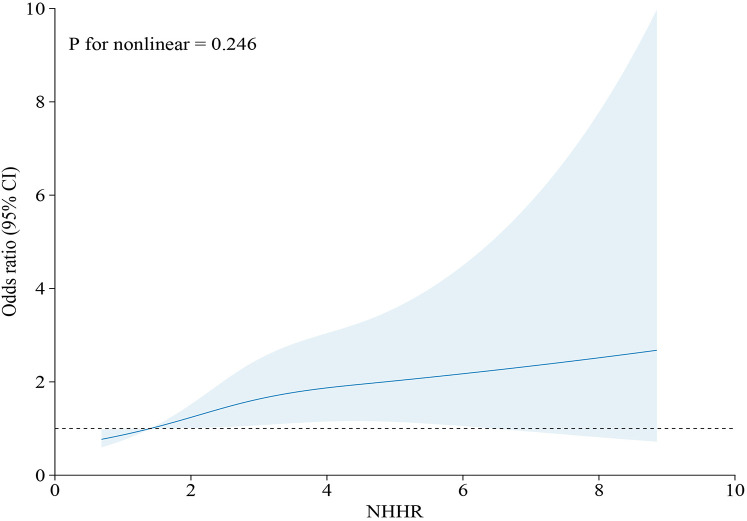
Restricted cubic spline model depicting the relationship between NHHR and sAAC. The restricted cubic spline model was adjusted for age, gender, race, BMI, marital status, education, PIR, smoking history, drinking history, hypertension, diabetes, heart failure, coronary heart disease, angina pectoris, stroke, total cholesterol, creatinine, serum calcium, phosphorus, uric acid, total 25-hydroxyvitamin D, and glycated hemoglobin.

Restricted cubic spline analyses (Figures 2, 3) revealed a monotonic dose-response association between NHHR concentrations and AAC scores (*p* for nonlinearity = 0.614). In the comprehensive adjusted model, NHHR concentrations demonstrated a significant positive linear correlation with sAAC (*p* for nonlinearity = 0.246).

### 3.5 Subgroup analysis

**Figure 4:**
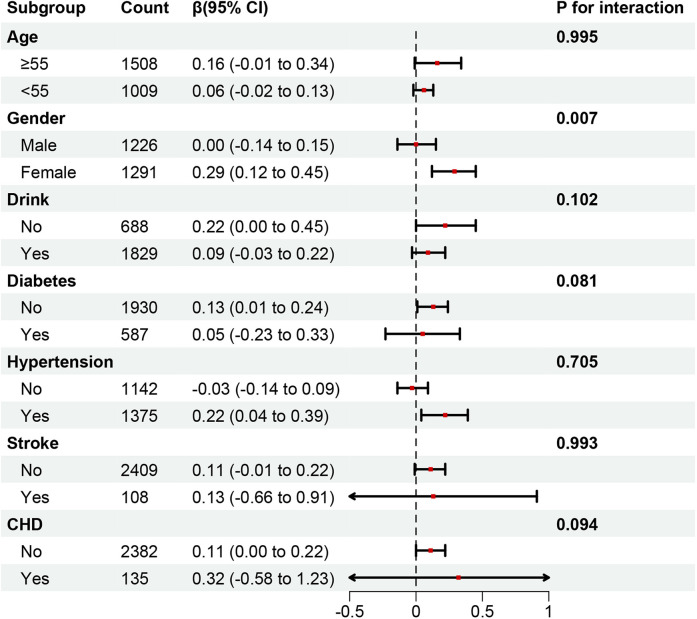
Subgroup analysis of the association between NHHR as a continuous variable and AAC score. In addition to the corresponding stratification variables, Model 3 was adjusted for age, gender, race, BMI, marital status, education, PIR, smoking history, drinking history, hypertension, diabetes, heart failure, coronary heart disease, angina pectoris, stroke, total cholesterol, creatinine, serum calcium, phosphorus, uric acid, total 25-hydroxyvitamin D, and glycated hemoglobin.

**Figure 5:**
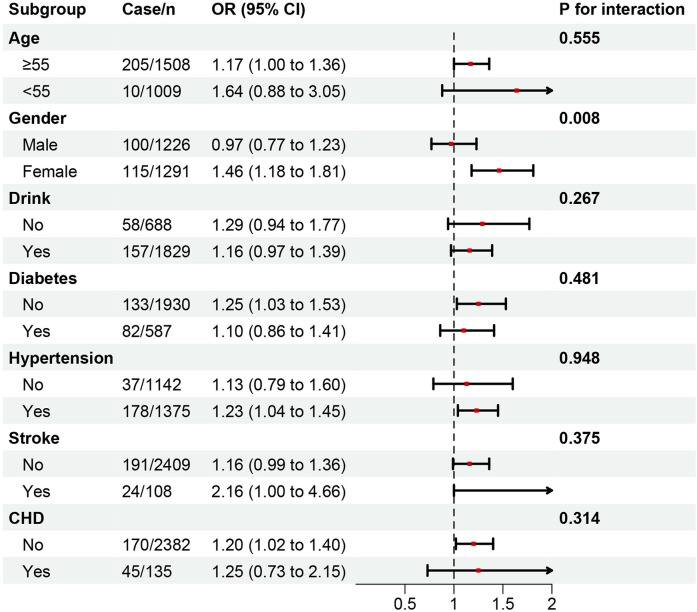
Subgroup analysis of the association between NHHR as a continuous variable and sAAC. In addition to the corresponding stratification variables, Model 3 was adjusted for age, gender, race, BMI, marital status, education, PIR, smoking history, drinking history, hypertension, diabetes, heart failure, coronary heart disease, angina pectoris, stroke, total cholesterol, creatinine, serum calcium, phosphorus, uric acid, total 25-hydroxyvitamin D, and glycated hemoglobin.

Subgroup analyses, in which NHHR was treated as a continuous variable and fully adjusted for covariates, are presented in Figures 4, 5. Across all subgroups stratified by age, sex, drinking status, and history of underlying diseases (diabetes, hypertension, stroke, and CHD), NHHR was consistently positively correlated with AAC (both AAC score and sAAC). No significant interactions were observed between NHHR and other subgroup variables, except for gender, where a significant interaction was detected [*p* for interaction (OR) = 0.007; *p* for interaction (*β*) = 0.008]. Meanwhile, the positive association between NHHR and the severity of AAC remained consistent across different racial groups (Supplementary Tables 10–12).

## 4 Discussion

This cross-sectional study examined the association between NHHR and AAC in U.S. adults aged 40 years and older. The findings indicate that, after adjusting for demographic factors, renal function, bone metabolism markers, and cardiovascular risk factors, NHHR was positively correlated with both AAC score and sAAC risk. Subgroup analysis revealed that this relationship remained consistent across various subgroups, suggesting its relative stability within the general population.

As a novel lipid-based index for assessing atherosclerosis, NHHR has demonstrated clinical predictive value for angina (13) and CHD (14) in previous studies. Vascular calcification, an actively regulated process that occurs early in atherosclerotic lesions (15), serves as an important predictor of cardiovascular events. AAC, a recognized marker of subclinical atherosclerosis, is also an independent predictor of cardiovascular event and mortality (16). Investigating the relationship between NHHR and AAC is therefore crucial for the early detection of subclinical atherosclerosis and cardiovascular risk. According to Matthew et al. (17), HDL-C demonstrates the strongest correlation with the severity of abdominal aortic sclerosis within the lipid profile. Additionally, Data from the NHANES study (18) demonstrated a positive association between elevated residual cholesterol (RC) levels and increased risk of sAAC in women. By integrating both atherogenic and anti-atherogenic lipid parameters, NHHR provides a more comprehensive measure for assessing AAC presence and severity.

In this study, after stepwise regression adjustment for demographic, lifestyle, and metabolic confounding factors, the association between NHHR and AAC was revealed, demonstrating an independent positive relationship between the two. Although the effect size of NHHR on AAC was relatively modest, the association remained statistically significant and consistent across fully adjusted models, indicating a certain degree of robustness. Furthermore, given the racial diversity of the NHANES population, we further explored the potential impact of race on this association. Despite variations in the prevalence and severity of AAC among different racial groups, the positive relationship between NHHR and AAC remained consistent across all subgroups. This suggests that, although genetic background, dietary habits, and socioeconomic factors may contribute to baseline differences among populations, the biological link between lipid metabolism abnormalities reflected by NHHR and vascular calcification is relatively stable and not dependent on racial differences. Therefore, NHHR may serve as a potential cross-ethnic indicator for assessing the risk of vascular calcification and atherosclerosis. From a clinical perspective, the relatively small effect size suggests that NHHR alone may have limited predictive utility for individual risk assessment. However, from a population-wide and multi-ethnic standpoint, even a modest increase in relative risk could translate into a notable increase in absolute case numbers, carrying important public health implications. Nevertheless, as this was a cross-sectional study, the actual predictive value of NHHR warrants further validation in prospective studies.

The development of AAC is influenced by multiple pathological mechanisms, including metabolic disorders and inflammatory responses. Based on existing literature, this study proposes the following potential mechanism underlying NHHR's effect on AAC. First, NHHR may be linked to AAC through lipid metabolism dysregulation. As the ratio of non-HDL-C to HDL-C, an elevated NHHR value is strongly associated with lipid metabolic disorders. Specifically, a high NHHR is often associated with elevated non-HDL-C and reduced HDL-C concentrations. Among non-HDL components, oxidized low-density lipoprotein (ox-LDL) plays a crucial role in stimulating the transformation of vascular smooth muscle cells (VSMCs) into osteoblast-like cells, thereby promoting vascular medial calcification (19). HDL-C exerts anti-inflammatory, antioxidant, and anti-atherosclerotic effects. Additionally, it facilitates cholesterol efflux and reverse cholesterol transport, which are critical processes for mitigating atherosclerosis and vascular calcification (20). These findings support the positive correlation between high NHHR and AAC severity. Although the precise mechanism linking lipid metabolism disorders to AAC remains unclear, managing NHHR as a comprehensive lipid index may help mitigate AAC progression and reduce the risk of cardiovascular events.

Another potential mechanism underlying AAC progression is the inflammatory response. Studies have shown that inflammatory gene expression in macrophages within foam cells is elevated in advanced atherosclerotic plaques compared to regressing plaques (21), supporting the hypothesis that lipids contribute to AAC progression through inflammatory pathways. In recent years, several inflammatory signaling pathways involving low-density lipoprotein (LDL) and macrophages in atherosclerotic plaques have been identified, including the TLR2, TLR4, and MYD88 pathways, which regulate cytokine gene expression and influence atherosclerosis development (22–24). Additionally, ox-LDL has been shown to activate the TLR4-TLR6-CD36 pathway, triggering inflammasomes to induce the secretion of significant quantities of pro-inflammatory cytokines, including interleukin-1β (IL-1β) and interleukin-18 (IL-18) (20). IL-1β, in particular, regulates inflammatory signaling cascades in VSMCs via the NF-kappa B and WNT/β-catenin pathways (25, 26), thereby promoting vascular calcification. Similarly, IL-8 has been found to induce the differentiation of circulating osteoblasts, further contributing to vascular calcification (19). Beyond lipid-driven inflammation, other inflammatory factors also influence AAC. Pro-inflammatory diets (27) and pan-immune inflammation values (PIV) have been implicated in vascular calcification. An NHANES study reported a positive correlation between PIV and AAC (28), reinforcing the strong association between inflammation and AAC. Although the precise role of inflammation in AAC progression across different stages remains unclear, the mechanisms outlined above warrant further investigation. Furthermore, due to the cross-sectional design of this study, the possibility of reverse causation cannot be entirely excluded, meaning that individuals with AAC may themselves exhibit lipid metabolism abnormalities. In addition, potential measurement errors in lipid profiles or AAC may have partially attenuated or overestimated the true strength of the association. Therefore, further validation through longitudinal and interventional studies is warranted to confirm these findings and to elucidate the causal mechanisms underlying the relationship between NHHR and AAC.

To further investigate whether the relationship between NHHR and AAC is influenced by gender, alcohol consumption, age, diabetes, CHD, stroke, and hypertension, a subgroup analysis was conducted. The interaction between NHHR and these factors was examined, revealing a positive correlation between NHHR and AAC across all subgroups. However, in the gender subgroup, NHHR was negatively correlated with AAC in males, potentially due to the role of cholesterol as a steroid hormone. This difference may be associated with gender-specific hormone levels (29). Androgens have been shown to directly stimulate Gas6 expression in VSMCs, preventing apoptosis (30) and thereby inhibiting vascular calcification. A recent NHANES study also reported a significant interaction between residual cholesterol (RC) and sAAC in gender subgroups, demonstrating that elevated RC concentrations in female participants independently predicted higher sAAC incidence rates (18) consistent with our findings.

This study has several notable strengths. First, it is the first to investigate the relationship between NHHR, AAC scores, and sAAC in a nationally representative sample of U.S. adults. Second, the findings are reliable, as the study followed standardized protocols and controlled for potential confounding variables based on previous research. However, several limitations should be acknowledged. As a cross-sectional study, causal relationships among NHHR, AAC scores, and sAAC cannot be established, highlighting the need for confirmation in prospective studies. Additionally, this study did not include younger individuals or pregnant women and was conducted only in adults aged 40 years and older. Therefore, the generalizability of our findings to the entire population still requires prospective validation in different age groups and specific populations. Moreover, smoking and drinking status were defined based on lifetime consumption, which may limit the precise assessment of lifestyle-related risk factors in relation to AAC; future studies with larger sample sizes and more detailed classification may help address this potential confounding. Finally, in addition to the biological mechanisms proposed in this study, reverse causation and random errors in data measurement may influence the true strength of the observed associations.

## 5 Conclusion

In adults aged 40 years and older, NHHR levels were positively associated with AAC scores and the risk of sAAC. This finding highlights the potential clinical value of NHHR in predicting the severity of AAC and the risk of cardiovascular complications.

## Data Availability

Publicly available datasets were analyzed in this study. This data can be found here: https://wwwn.cdc.gov/nchs/nhanes/.
