# Supplementary material for: The relationship between non-high-density lipoprotein cholesterol to high-density lipoprotein cholesterol ratio and abdominal aortic calcification in adults: a cross-sectional study
Source: Front Cardiovasc Med. 2025 Nov 17;12:1578407. doi: 10.3389/fcvm.2025.1578407 (PMC12665718; doi:10.3389/fcvm.2025.1578407)
Supplement: Supplementary file 1 [file Datasheet1.pdf]

frontiers

Supplementary Material

Supplementary Table 1. Abdominal Aortic Calcification (AAC) Scoring Criteria

Grade	Evaluation standard
0	No calcification；
1	Less than 1/3 calcification；
2	Calcification is greater than 1/3 and less than 2/3;
3	Calcification is more than 2/3；

This method divides the anterior and posterior walls of the aorta into eight segments aligned with the L1–L4 vertebrae. Points are assigned to the anterior and posterior walls, with each section scored from 0 to 3, resulting in a total possible score ranging from 0 to 24.

Supplementary Table 2. Multivariable logistic regression analysis of the association between NHHR and AAC score (Model 2).

	Odds Ratio (OR)	Standard Error (SE)	95% CI (Lower Limit)	95% CI (Upper Limit)	*p* value
(Intercept)	0.022	0.503	0.008	0.058	<0.001
NHHR	1.090	0.046	0.995	1.193	0.063
Gender (Female)	1.113	0.124	0.872	1.419	0.390
Age	1.110	0.005	1.099	1.122	< 0.001
Race (Other Hispanic)	0.834	0.265	0.497	1.400	0.492
Race (Non-Hispanic White)	1.590	0.196	1.083	2.335	0.018
Race (Non-Hispanic Black)	0.771	0.222	0.499	1.190	0.240
Race (Other)	0.992	0.247	0.6115	1.6106	0.975404
BMI	0.957	0.012	0.936	0.979	< 0.001

Supplementary Table 3. Multivariable logistic regression analysis of the association between NHHR and AAC score (Model 3).

	Odds Ratio (OR)	Standard Error (SE)	95% CI (Lower Limit)	95% CI (Upper Limit)	*p* value
(Intercept)	0.017	1.663	0.001	0.437	0.014
NHHR	1.137	0.057	1.018	1.270	0.023
Gender (Female)	1.455	0.150	1.085	1.951	0.012
Age	1.082	0.006	1.070	1.095	< 0.001
Marital status (Widowed/Divorced/Separated)	1.507	0.143	1.139	1.994	0.004
Marital status (Unmarried)	0.947	0.232	0.602	1.492	0.816
Race (Other Hispanic)	0.795	0.259	0.478	1.322	0.378
Race (Non-Hispanic White)	1.507	0.204	1.010	2.249	0.045
Race (Non-Hispanic Black)	0.613	0.228	0.392	0.959	0.032
Race (Other)	0.997	0.253	0.608	1.637	0.992
Education level (High school or equivalent)	1.208	0.185	0.840	1.736	0.309
Education level (Above high school)	1.024	0.174	0.728	1.442	0.891
PIR	1.011	0.043	0.929	1.100	0.801
BMI	0.930	0.012	0.908	0.953	< 0.001
Smoke (Yes)	1.524	0.130	1.182	1.964	0.001
Glycohemoglobin	1.064	0.067	0.933	1.214	0.355
Drink (Yes)	1.129	0.151	0.840	1.518	0.420

Hypertension (Yes)	1.569	0.133	1.208	2.038	< 0.001
Diabetes (Yes)	1.579	0.186	1.096	2.274	0.014
Heart failure (Yes)	2.467	0.358	1.223	4.976	0.012
CHD (Yes)	4.686	0.326	2.476	8.870	< 0.001
Angina (Yes)	0.882	0.372	0.426	1.828	0.736
Heart attack (Yes)	1.541	0.311	0.837	2.839	0.165
Stroke (Yes)	1.649	0.299	0.917	2.963	0.095
TC	0.997	0.002	0.993	1.000	0.054
Creatinine	1.173	0.121	0.925	1.487	0.188
Serum calcium	1.125	0.169	0.808	1.566	0.486
Serum phosphorus	1.093	0.110	0.880	1.356	0.422
Uric acid	1.053	0.050	0.954	1.162	0.306
Total 25-hydroxyvitamin D	0.998	0.002	0.993	1.002	0.283

Supplementary Table 4. Multivariable logistic regression analysis of the association between quartiles of NHHR and AAC score (Model 2).

	Odds Ratio (OR)	Standard Error (SE)	95% CI (Lower Limit)	95% CI (Upper Limit)	*p* value
(Intercept)	0.025	0.495	0.009	0.065	< 0.001
NHHR(Q2)	1.038	0.173	0.740	1.458	0.828
NHHR(Q3)	1.130	0.177	0.799	1.599	0.490
NHHR(Q4)	1.400	0.182	0.981	2.000	0.064
Gender (Female)	1.116	0.124	0.875	1.424	0.377
Age	1.110	0.005	1.099	1.122	< 0.001
Race (Other Hispanic)	0.839	0.265	0.500	1.409	0.507
Race (Non-Hispanic White)	1.604	0.196	1.092	2.355	0.016
Race (Non-Hispanic Black)	0.776	0.222	0.502	1.200	0.255
Race (Other)	0.998	0.247	0.615	1.621	0.995
BMI	0.957	0.012	0.935	0.979	< 0.001

Supplementary Table 5. Multivariable logistic regression analysis of the association between quartiles of NHHR and AAC score (Model 3).

	Odds Ratio (OR)	Standard Error (SE)	95% CI (Lower Limit)	95% CI (Upper Limit)	*p* value
(Intercept)	0.020	1.667	0.001	0.518	0.019
NHHR (Q2)	1.106	0.171	0.790	1.547	0.557
NHHR (Q3)	1.238	0.181	0.868	1.765	0.238
NHHR (Q4)	1.551	0.209	1.029	2.336	0.036
Gender (Female)	1.432	0.149	1.070	1.917	0.016
Age	1.082	0.006	1.069	1.094	<0.001
Marital status (Widowed/Divorced/Separated)	1.504	0.143	1.137	1.991	0.004
Marital status (Unmarried)	0.953	0.232	0.605	1.502	0.837
Race (Other Hispanic)	0.802	0.260	0.482	1.335	0.397
Race (Non-Hispanic White)	1.531	0.204	1.026	2.284	0.037
Race (Non-Hispanic Black)	0.619	0.229	0.395	0.969	0.036
Race (Other)	1.008	0.253	0.614	1.655	0.975
Education level (High school or equivalent)	1.206	0.185	0.838	1.734	0.313
Education level (Above high school)	1.021	0.175	0.725	1.438	0.903
PIR	1.009	0.043	0.928	1.099	0.828
BMI	0.931	0.012	0.909	0.953	<0.001

Smoke (Yes)	1.526	0.130	1.184	1.968	0.001
Glycohemoglobin	1.067	0.067	0.936	1.217	0.332
Drink (Yes)	1.126	0.151	0.838	1.514	0.432
Hypertension (Yes)	1.568	0.133	1.207	2.037	0.001
Diabetes (Yes)	1.588	0.186	1.102	2.288	0.013
Heart failure (Yes)	2.480	0.358	1.229	5.004	0.011
CHD (Yes)	4.676	0.326	2.469	8.856	<0.001
Angina (Yes)	0.890	0.372	0.429	1.845	0.753
Heart attack (Yes)	1.535	0.312	0.833	2.827	0.169
Stroke (Yes)	1.651	0.299	0.918	2.967	0.094
TC	0.997	0.002	0.994	1.000	0.080
Creatinine	1.175	0.121	0.926	1.490	0.185
Serum calcium	1.117	0.169	0.802	1.555	0.513
Serum phosphorus	1.092	0.110	0.880	1.356	0.425
Uric acid	1.049	0.051	0.950	1.158	0.348
Total 25-hydroxyvitamin D	0.998	0.002	0.993	1.002	0.284

Supplementary Table 6. Multivariable logistic regression analysis of the association between NHHR and sAAC (Model 2).

	Odds Ratio (OR)	Standard Error (SE)	95% CI (Lower Limit)	95% CI (Upper Limit)	*p* value
(Intercept)	0.000	0.800	0.000	0.001	< 0.001
NHHR	1.097	0.060	0.975	1.234	0.124
Gender (Female)	1.162	0.153	0.861	1.569	0.326
Age	1.122	0.008	1.103	1.141	< 0.001
Race (Other Hispanic)	0.557	0.428	0.241	1.287	0.171
Race (Non-Hispanic White)	1.262	0.277	0.734	2.171	0.400
Race (Non-Hispanic Black)	0.709	0.333	0.369	1.362	0.302
Race (Other)	0.964	0.353	0.483	1.924	0.916
BMI	0.959	0.016	0.930	0.990	0.009

Supplementary Table 7. Multivariable logistic regression analysis of the association between NHHR and sAAC (Model 3).

	Odds Ratio (OR)	Standard Error (SE)	95% CI (Lower Limit)	95% CI (Upper Limit)	*p* value
(Intercept)	0.000	2.381	0.000	0.001	< 0.001
NHHR	1.193	0.078	1.024	1.389	0.023
Gender (Female)	1.343	0.215	0.881	2.047	0.171
Age	1.106	0.010	1.084	1.129	< 0.001
Marital status (Widowed/Divorced/Separated)	1.586	0.191	1.092	2.305	0.016
Marital status (Unmarried)	0.914	0.425	0.398	2.101	0.833
Race (Other Hispanic)	0.562	0.473	0.223	1.419	0.223
Race (Non-Hispanic White)	1.388	0.329	0.728	2.645	0.319
Race (Non-Hispanic Black)	0.639	0.382	0.302	1.353	0.242
Race (Other)	1.068	0.406	0.482	2.368	0.871
Education level (High school or equivalent)	0.870	0.253	0.530	1.427	0.580
Education level (Above high school)	0.807	0.241	0.503	1.294	0.374
PIR	1.089	0.063	0.962	1.233	0.176
BMI	0.933	0.019	0.898	0.968	0.000
Smoke (Yes)	1.953	0.187	1.355	2.816	0.000
Glycohemoglobin	1.140	0.083	0.969	1.341	0.115
Drink (Yes)	1.285	0.214	0.845	1.955	0.242

Hypertension (Yes)	2.022	0.214	1.330	3.074	0.001
Diabetes (Yes)	1.437	0.230	0.917	2.254	0.114
Heart failure (Yes)	1.490	0.344	0.759	2.925	0.247
CHD (Yes)	2.235	0.307	1.225	4.077	0.009
Angina (Yes)	1.004	0.382	0.475	2.124	0.991
Heart attack (Yes)	0.851	0.325	0.451	1.608	0.619
Stroke (Yes)	1.245	0.296	0.697	2.226	0.459
TC	0.995	0.003	0.990	1.000	0.042
Creatinine	1.061	0.139	0.808	1.392	0.671
Serum calcium	1.161	0.223	0.750	1.799	0.503
Serum phosphorus	1.457	0.159	1.066	1.992	0.018
Uric acid	0.975	0.066	0.857	1.109	0.700
Total 25-hydroxyvitamin D	1.000	0.003	0.994	1.005	0.919

Supplementary Table 8. Multivariable logistic regression analysis of the association between quartiles of NHHR and sAAC (Model 2).

	Odds Ratio (OR)	Standard Error (SE)	95% CI (Lower Limit)	95% CI (Upper Limit)	*p* value
(Intercept)	0.000	0.787	0.000	0.001	< 0.001
NHHR(Q2)	1.145	0.206	0.765	1.713	0.512
NHHR(Q3)	1.156	0.218	0.754	1.771	0.506
NHHR(Q4)	1.474	0.224	0.951	2.286	0.083
Gender (Female)	1.163	0.153	0.862	1.571	0.323
Age	1.122	0.008	1.103	1.140	< 0.001
Race (Other Hispanic)	0.556	0.427	0.241	1.286	0.170
Race (Non-Hispanic White)	1.271	0.277	0.739	2.186	0.386
Race (Non-Hispanic Black)	0.713	0.333	0.371	1.370	0.310
Race (Other)	0.966	0.352	0.484	1.927	0.922
BMI	0.958	0.016	0.929	0.989	0.008

Supplementary Table 9. Multivariable logistic regression analysis of the association between quartiles of NHHR and sAAC (Model 3).

	Odds Ratio (OR)	Standard Error (SE)	95% CI (Lower Limit)	95% CI (Upper Limit)	*p* value
(Intercept)	0.000	2.388	0.000	0.001	< 0.001
NHHR (Q2)	1.345	0.233	0.851	2.124	0.204
NHHR (Q3)	1.612	0.255	0.979	2.657	0.061
NHHR (Q4)	1.959	0.293	1.102	3.482	0.022
Gender (Female)	1.330	0.214	0.874	2.025	0.183
Age	1.106	0.010	1.084	1.129	< 0.001
Marital status (Widowed/Divorced/Separated)	1.589	0.191	1.094	2.309	0.015
Marital status (Unmarried)	0.944	0.423	0.412	2.162	0.892
Race (Other Hispanic)	0.559	0.472	0.221	1.411	0.219
Race (Non-Hispanic White)	1.419	0.329	0.744	2.705	0.288
Race (Non-Hispanic Black)	0.647	0.383	0.306	1.372	0.257
Race (Other)	1.088	0.406	0.491	2.410	0.835
Education level (High school or equivalent)	0.857	0.253	0.522	1.408	0.543
Education level (Above high school)	0.797	0.241	0.497	1.277	0.345
PIR	1.091	0.063	0.964	1.234	0.169
BMI	0.931	0.019	0.897	0.967	0.000

Smoke (Yes)	1.948	0.186	1.352	2.807	0.000
Glycohemoglobin	1.143	0.083	0.971	1.344	0.108
Drink (Yes)	1.295	0.215	0.850	1.973	0.228
Hypertension (Yes)	2.031	0.214	1.335	3.089	0.001
Diabetes (Yes)	1.442	0.230	0.919	2.262	0.111
Heart failure (Yes)	1.511	0.345	0.769	2.969	0.232
CHD (Yes)	2.223	0.306	1.220	4.054	0.009
Angina (Yes)	1.038	0.382	0.490	2.195	0.923
Heart attack (Yes)	0.857	0.324	0.454	1.615	0.632
Stroke (Yes)	1.257	0.296	0.703	2.245	0.441
TC	0.995	0.003	0.990	1.000	0.045
Creatinine	1.064	0.139	0.811	1.397	0.653
Serum calcium	1.158	0.223	0.748	1.794	0.511
Serum phosphorus	1.473	0.160	1.077	2.015	0.015
Uric acid	0.965	0.066	0.847	1.098	0.586
Total 25-hydroxyvitamin D	1.000	0.003	0.994	1.005	0.941

Supplementary Table 10. Comparison of NHHR Levels and Severity of AAC Among Different Race Groups.

	Level	Mexican American	Other Hispanic	Non-Hispanic White	Non-Hispanic Black	Other	*p*
Overall		312	231	1,172	483	319	
NHHR (mean (SD))		3.15 (1.31)	3.12 (1.44)	2.87 (1.44)	2.62 (1.33)	2.97 (1.32)	<0.001 [b]
AAC score (mean (SD))		1.04 (2.46)	1.08 (2.71)	2.07 (3.91)	1.01 (2.61)	1.21 (2.70)	<0.001 [b]
sAAC (%)	No	295 (94.6)	222 (96.1)	1027 (87.6)	459 (95.0)	299 (93.7)	<0.001 [a]
	Yes	17 (5.4)	9 (3.9)	145 (12.4)	24 (5.0)	20 (6.3)	

[a] : P value calculated using the Chi-square test. [b] : P value calculated using the Kruskal–Wallis test.

Supplementary Table 11. Subgroup Analysis of the Association Between NHHR and AAC Score Across Different Race Groups.

Variable	Count	Percent	β	Lower	Upper	*p* value	*p* for interaction
Overall	2517	100	0.13	0.02	0.24	0.023	
Race							**0.802**
Mexican American	312	12.4	-0.18	-0.46	0.1	0.217	
Other Hispanic	231	9.2	-0.04	-0.36	0.27	0.801	
Non-Hispanic White	1172	46.6	0.16	-0.02	0.34	0.082	
Non-Hispanic Black	483	19.2	0.11	-0.11	0.33	0.337	
Other	319	12.7	0.16	-0.12	0.43	0.262	

Supplementary Table 12. Subgroup Analysis of the Association Between NHHR and sAAC Across Different Race Groups.

Variable	Count	Percent	OR	Lower	Upper	*p* value	*p* for interaction
Overall	2517	100	1.19	1.03	1.38	0.02	
Race							**0.383**
Mexican American	312	12.4	0.67	0.29	1.56	0.349	
Other Hispanic	231	9.2	0	0	Inf	1	
Non-Hispanic White	1172	46.6	1.22	1.01	1.48	0.039	
Non-Hispanic Black	483	19.2	1.37	0.84	2.24	0.207	
Other	319	12.7	0.62	0.19	2.07	0.44	
